# The Importance of HbA1c and Left Ventricular Ejection Fraction in Predicting the Development of Postoperative Mortality and Complications in Coronary Artery Bypass Graft Surgery

**DOI:** 10.21470/1678-9741-2020-0542

**Published:** 2022

**Authors:** Rifat Özmen, Aydın Tunçay, Halis Yılmaz, Gülden Sarı, Haluk Kutay Taşdemir

**Affiliations:** 1 Department of Cardiovascular Surgery, Erciyes University School of Medicine, Kayseri, Turkey.

**Keywords:** Coronary Artery Bypass Grafts, CABG, Hba1c, Diabetes Mellitus, Surgery, Complications.

## Abstract

**Introduction:**

In this study, we aimed to investigate the relationship between postoperative mortality, morbidity, hospital stay and development of postoperative complications with the glycosylated hemoglobin (HbA1c) level and low left ventricular ejection fraction (LVEF) in diabetic and non-diabetic patients who underwent elective coronary artery bypass (CABG) surgery.

**Methods:**

The medical records of patients who underwent CABG at our clinic between January 2015 and December 2019 were retrospectively analyzed. All patients were divided into two groups according to their diabetes mellitus (DM) diagnosis. Diabetic patients were also divided into two groups according to their HbA1c levels. The HbA1c threshold value was 7%. All patients were divided into two groups in terms of LVEF. The LVEF threshold value was 40%.

**Results:**

We analyzed 393 patients, of which 304 (77.4%) were male and 177 (45.04%) patients were diabetic. For lower LVEF and HbA1c values, we found no relationship between postoperative mortality, prolonged intensive care unit (ICU) stay and development of postoperative complications. Deep surgical site infection (DSSI) was found to be more common in diabetic patients who had a higher HbA1c value. Length of hospital stay was longer in diabetic patients with HbA1c levels <7%.

**Conclusion:**

No statistically significant relationship was found between LVEF and HbA1c levels and postoperative mortality, prolonged ICU stay and postoperative complications.

**Table:** 

Abbreviations, acronyms & symbols				
AF	= Atrial fibrillation		DSSI	= Deep surgical site infection
AKI	= Acute kidney injury			
AMI	= Acute myocardial infarction		EF	= Ejection fraction
BSA	= Body surface area		HbA1c	= Glycosylated hemoglobin
CABG	= Coronary artery bypass graft		HF	= Heart failure
CAD	= Carotid artery disease		ICU	= Intensive care unit
COPD	= Chronic obstructive pulmonary disease		LIMA	= Left internal mammary artery
CPB	= Cardiopulmonary bypass		LVEF	= Left ventricular ejection fraction
CKD	= Chronic kidney disease		LV	= Left ventricle
CVD	= Cerebrovascular disease		PCI	= Percutaneous coronary intervention
DM	= Diabetes mellitus		XCL	= Cross-clamp

## INTRODUCTION

It is known that approximately 425 million people worldwide have type II diabetes, and the prevalence of coronary artery disease in diabetic patients is quite high. In general, vascular diseases involving coronary heart and cerebrovascular diseases account for 65% of all deaths in diabetic patients with multiple coronary vascular diseases and coronary artery bypass graft (CABG) surgery is preferred as the primary treatment^[1]^. Therefore, preoperative risk analysis is very important in predicting postoperative results in diabetic patients undergoing open-heart surgery^[2]^.

Ineffective glycemic control in the perioperative period of coronary bypass surgery in diabetic patients is known to negatively affect the outcomes that may occur in the postoperative period^[3]^. Furthermore, it has been reported that postoperative results are better in diabetic patients who had effective glycemic control during the perioperative period^[4]^.

The glycosylated hemoglobin (HbA1c) level is commonly used to diagnose diabetes and to measure the effectiveness of antidiabetic therapy, and HbA1c threshold is one of the parameters frequently studied in diabetic patients in terms of estimation of postoperative outcomes of open-heart surgery^[2]^. Currently, there are several studies showing that HbA1c is associated with prolonged hospital stay, morbidity and mortality in diabetic patients undergoing CABG^[9^,^10]^. On the other hand, there are also some studies that argue about the no relationship between them^[1^,^2^,^5^-^8]^. In the diabetic patient group, HbA1c levels ≥7% indicate poor glycemic control and help to predict postoperative complications for CABG surgery^[2]^. Najafi et al.^[9]^ and Medhi et al.^[10]^ have shown that HbA1c was significantly associated with postoperative hospital stay. Contrary to the above, a study conducted by Almogati et al.^[2]^ with 305 patients found that high HbA1c levels did not affect the hospital stay in diabetic patients undergoing CABG. Khan et al.^[11]^ found a high risk of mortality and postoperative infections in patients with systolic heart failure. In another study, Aydınlı et al.^[1]^ found no relationship between high HbA1c levels and postoperative mortality and morbidity, but reported that low left ventricular ejection fraction (LVEF) is a high-risk factor for mortality and morbidity independent of HbA1c levels in these patients.

In this study, we aimed to investigate whether the presence of diabetes, HbA1c levels in diabetic and non-diabetic patients and low LVEF were risk factors for postoperative mortality, hospital stay and development of postoperative complications on patients undergoing elective CABG.

## METHODS

After approval by the Ethics Committee of the Erciyes University Faculty of Medicine (26.02.2020 and number 2020/138), medical records of sequential patients undergoing isolated elective coronary bypass surgery at our clinic between January 2015 and December 2019 were analyzed retrospectively. This study included patients aged 18 to 80 years who underwent elective on-pump CABG. We excluded patients in the following conditions: (i) those who underwent emergency coronary bypass; (ii) those who underwent a combined procedure; (iii) those who underwent off-pump heart surgery; and (iv) those with a history of coronary bypass surgery. After applying exclusion criteria, 393 patients were included in the study.

Demographic information of the patients, comorbidity conditions in the preoperative period, cross-clamp (XCL) times, cardiopulmonary bypass (CPB) times, LVEF, intensive care unit (ICU) and hospital stays, types of grafts used, number of distal anastomoses, body surface area (BSA) and HbA1c levels were recorded.

All patients were divided into two groups: those with a diagnosis of diabetes mellitus (DM) and those without a diagnosis of DM. The patients diagnosed with DM were also divided into two groups: those with HbA1c <7% and those with HbA1c ≥7%. The HbA1c threshold value was 7%, which was reported as most appropriate for evaluating high-risk groups^[2]^.

Since it was estimated that 40% of hospitalized patients with heart failure (HF) with reduced LVEF have DM, the etiology of HF encompasses many different conditions, yet coronary heart disease and acute myocardial infarction (AMI) were considered among the most frequent underlying causes. For this reason, we evaluated if low LVEF was a risk factor for postoperative mortality, morbidity, prolonged hospital stay and development of postoperative complications on patients undergoing elective CABG.

All patients were divided into two groups in terms of LVEF. LVEF threshold value was 40%. Shortly, LV diameters and wall thickness were measured (according to the American Society of Echocardiography guidelines) from parasternal views by M-mode echocardiography. Simpson’s method was used to calculate LVEF. The area and diameter of the left atrium were measured in the parasternal long-axis view. All measurements were made by the same team members.

Postoperative complications were considered as acute kidney injury (AKI), cerebrovascular disease (CVD), atrial fibrillation (AF) and deep surgical site infection (DSSI).

For postoperative AKI, the definition of KDIGO^[12]^ (serum creatinine increased by 0.3 mg/dL in the first 48 hours according to preoperative value or ≥ 1.5 times increase in 3 days after cardiac surgery) was used.

Postoperative CVD in patients without preoperative neurological findings, any temporary or permanent loss of strength, speech impairment or postoperative visual loss were considered as events diagnosed clinically and radiologically by the neurologist.

AF in the patient with a preoperative normal sinus rhythm was considered to have no postoperative P wave on the electrocardiogaphy at admission, and was determined as an arrhythmia detected by the irregularity of the distance between R-R intervals.

DSSI was accepted as a condition that requires debridement, drainage and antibiotics in the sternal area, growth of the microorganism in culture and diagnosis by an infectious disease specialist.

### Statistical Analysis

If the data distribution was normal, it was evaluated by the Shapiro-Wilk test. The Mann-Whitney U test was used to compare the sociodemographic and clinical data of the patient groups (since the data did not have a normal distribution). Categorical data of patient groups such as female/male ratio, presence of additional disease and mortality rate were compared with the chi-square test. The Spearman correlation test was applied to investigate the relationship between clinical data, numerical variables in the tables were expressed as mean±standard deviation and categorical variables as percentages. Statistical significance level was accepted as *P*<0.05.

## RESULTS

In this study, we analyzed a total of 393 patients, 304 (77.4%) males and 89 (22.6%) females. There was a diagnosis of DM in 177 (45.04%) of all patients.

The patients were divided into two groups: diabetic and non-diabetic. In the diabetic group, 132 patients were male, while 45 patients were female. There was no statistically significant difference between patients in terms of gender (χ^2^=1.418, *P*=0.234). The mean age was 63.85±9.09 in diabetic patients, while the mean age was 62.26±10.59 in non-diabetic patients. There was no statistically significant difference between the two groups in terms of age (Z=1.280, *P*=0.201). LVEF values in diabetic and non-diabetic patients were 51.52±7.250 and 51.55±7.248, respectively. For the LVEF value, there was no statistical difference between the groups (Z=0.209, *P*=0.834). The preoperative HbA1c values in non-diabetic and diabetic patients were 5.59±0.23 and 8.14±1.93 (Z=16.897, *P*<0.001) and were statistically higher in diabetic patients, respectively.

There was no significant difference between XCL time, CPB time and length of ICU and hospital stays in diabetic and non-diabetic patients (Table 1).

**Table 1:** Distribution of patients according to diabetic status.

	Diabetic (n=216)	Non-diabetic (n=177)	Comparison
HbA1c	5.59±0.232	8.142±1.938*	Z=16.897
			*P*<0.001
XCL time	64.07±26.511	61.05±22.871	Z=1.223
			*P*=0.221
CPB time	130.99±50.282	121,940±43.236	Z=0.587
			*P*=0.112
ICU stay	5.42±5.27	6.40±7.40	Z=0.568
			*P*=0.570
Length of hospital stay	13±8.749	13.898±9.642	Z=0.511
			*P*=0.609
Mortality (n=69, 17.6%)	30 (16.9%)	39 (18.1%)	χ^2^=0.082
			*P*=0.774
Postoperative CVD (n=7, 1.8%)	3 (1.4%)	4 (2.3%)	χ^2^=0.432
			*P*=0.511
Postoperative AF (n=117, 29.8%)	54 (30.7%)	63 (29.2%)	χ^2^=0.106
			*P*=0.744
Postoperative AKI (n=51, 13%)	26 (12%)	25 (14.2%)	χ^2^=0.403
			*P*=0.526
DSSI (n=6, 5.8%)	0	6 (10.5%)*	χ^2^=5.142
			*P*=0.0.23

AF=atrial fibrillation; AKI=acute kidney injury; CPB=cardiopulmonary bypass; DSSI=deep surgical site infection; HbA1c=glycosylated hemoglobin; ICU=intensive care unit; LIMA=left internal mammary artery; LVEF=left ventricular ejection fraction; XCL=cross-clamp

Hypertension and chronic kidney disease (CKD) were statistically higher in diabetic patients and pulmonary hypertension in non-diabetic patients (χ^2^=13.252, *P*<0.001, χ^2^=5.056, *P*=0.025, χ^2^=6.718, *P*=0.01). There was no significant difference between the groups in terms of preoperative chronic obstructive pulmonary disease (COPD), carotid artery disease (CAD) and intraoperative left internal mammary artery (LIMA) usage.

Mortality occured in 69 (17.6%) patients during hospitalization. Of these, 39 (18.1%) were non-diabetic and 30 (16.9%) were diabetic patients (χ^2^=0.082, *P*=0.774). DSSI (5.8%) developed in six patients (χ^2^=5.142, *P*=0.0.23). As our study was retrospective, morbidity rates of some patients were not available after discharge. For this reason, conditions causing morbidity were not evaluated. This is one of the limitations of our study.

For postoperative mortality, CVD, AF and AKI, there was no significant difference between diabetic and non-diabetic patients. DSSI developed only in diabetic patients’ group (χ^2^=5.142, *P*=0.023).

When the number of distal anastomoses was evaluated, eight patients had one (2%), 48 patients had two (12.2%), 137 patients had three (34.9%), 149 patients had four (37.9%), 42 patients had five (10.7%) and nine patients had six or more anastomoses (2.3%).

According to the LVEF, patients were divided into two groups to assess mortality, length of hospital and ICU stays, postoperative CVD, AKI and AF, and occurence of DSSI. We took the threshold below or above 40% for the LVEF value.

In 48 patients, LVEF was ≤40%. The mean LVEF was 37.67±3.191 in the group with ejection fraction (EF) ≤40%, and 53.47±5.268 in the group with EF >40%. There was a statistically significant difference between the two groups (Z=11.386, *P*<0.001).

There was no statistically significant difference between the groups in terms of postoperative mortality, CVD, AF, AKI, length of hospital and ICU stays, and DSSI (Table 2).

**Table 2:** Evaluation of patients according to LVEF values.

	EF <40 (n=48)	EF >40 (n=345)	Comparison
LVEF	37.67±3.191	53.47±5.268	Z=11.386
			*P*<0.001
ICU stay	4.833±4.710	6.012±6.517	Z=1.082
			*P*=0.279
Length of hospital stay	11.625±7.468	13.658±9.355	Z=1.277
			*P*=0.202
Mortality (n=69)	9 (18.8%)	60 (17.4%)	χ^2^=0.054
			*P*=0.817
Postoperative CVD (n=7)	2 (4.2%)	5 (1.5%)	χ^2^=1.768
			*P*=0.184
Postoperative AF (n=117)	18 (37.5%)	99 (28.8%)	χ^2^=1.530
			*P*=0.216
Postoperative AKI (n=51)	10 (20.8%)	41 (11.9%)	χ^2^=2.958
			*P*=0.085
DSSI (n=6)	0	6 (6.7%)*	χ^2^=0.920
			*P*=0.337

AF=atrial fibrillation; AKI=acute kidney injury; CPB=cardiopulmonary bypass; DSSI=deep surgical site infection; EF=ejection fraction; ICU=intensive care unit; LIMA=left internal mammary artery; LVEF=left ventricular ejection fraction; XCL=cross-clamp

All patients were divided into two groups according to their HbA1c levels. We took the threshold below or above 7% for the HbA1c level. There were 122 patients who had an HbA1c level ≥7%. Demographic data, presence of comorbidities, preoperative blood values, XCL time, CPB time, and LIMA usage were examined between the groups. The relationships between the HbA1c level and mortality, duration of hospital and ICU stays, postoperative CVD, AKI, AF and development of DSSI were evaluated. The results are summarized in Table 3.

**Table 3:** Evaluation of patients according to HbA1c levels.

	HbA1c <7% (n=271)	HbA1c >7% (n=122)	Comparison
HbA1c	5.753±0.420	8.935±1.831*	Z=15.930
			*P*<0.001
ICU stay	5.723±5.988	6.189±7.049	Z=0.475
			*P*=0.634
Length of hospital stay	13.413±8.825	13.402±9.904	Z=1.132
			*P*=0.258
Mortality (n=69)	49 (18.1%)	20 (16.4%)	χ^2^=0.166
			*P*=0.684
Postoperative CVD (n=7)	4 (1.5%)	3 (2.5%)	χ^2^=0.458
			*P*=0.499
Postoperative AF (n=117)	82 (30.4%)	35 (28.7%)	χ^2^=0.114
			*P*=0.736
Postoperative AKI (n=51)	35 (12.9%)	16 (13.2%)	χ^2^=0.007
			*P*=0.933
DSSI (n=6)	0	6 (13.6%)*	χ^2^=8.543
			*P*=0.003

AF=atrial fibrillation; AKI=acute kidney injury; CPB=cardiopulmonary bypass; DSSI=deep surgical site infection; HbA1c=glycosylated hemoglobin; ICU=intensive care unit; LIMA=left internal mammary artery; LVEF=left ventricular ejection fraction; XCL=cross-clamp

In patients who had a HbA1c level ≥7%, DSSI rates were higher than in patients with HbA1c levels <7%. There was no statistically significant difference between groups in terms of HbA1c levels, development of postoperative mortality, length of hospital and ICU stays, postoperative CVD, AKI and AF (Table 3).

Diabetic patients were also evaluated for their HbA1c levels and divided into two groups. The relationships between HbA1c level, XCL and CPB times, mortality, length of hospital and ICU stays, postoperative CVD, AKI, AF, and development of DSSI were evaluated. The results are presented in Table 4.

**Table 4:** Evaluation of diabetic patients according to HbA1c levels.

	Diabetic patients HbA1c <7 (n=55)	Diabetic patients HbA1c >7 (n=122)	Comparison
HbA1c	6.382±0.401	8.935±1.938*	Z=10.649
			*P*<0.001
XCL time	55.16±21.09	63.70±23.22*	Z=2.315
			*P*=0.021
CPB time	112.42±39.43	126.24±44.33*	Z=1.963
			*P*=0.05
ICU stay	6.89±8.17	6.18±7.04	Z=0.045
			*P*=0.964
Length of hospital stay	15±9.02	13.40±9.9*	Z=2.172
			*P*=0.03
Mortality (n=30)	10 (18.2%)	20 (16.4%)	χ^2^=0.086
			*P*=0.769
Postoperative CVD (n=4)	1 (1.9%)	3 (2.5%)	χ^2^=0.062
			*P*=0.803
Postoperative AF (n=54)	19 (35.2%)	35 (28.7%)	χ^2^=0.743
			*P*=0.389
Postoperative AKI (n=25)	9 (16.4%)	16 (13.2%)	χ^2^=0.306
			*P*=0.580
DSSI (n=6)	0	6 (13.6%)*	χ^2^=1.981
			*P*=0.159

AF=atrial fibrillation; AKI=acute kidney injury; CPB=cardiopulmonary bypass; DSSI=deep surgical site infection; HbA1c=glycosylated hemoglobin; ICU=intensive care unit; LIMA=left internal mammary artery; LVEF=left ventricular ejection fraction; XCL=cross-clamp

In diabetic patients whose HbA1c level was <7%, XCL and CPB times were shorter, and the length of their hospital stays was longer (Z=2.315, *P*=0.021, Z=1.963, *P*=0.05, Z=2.172, *P*=0.03) (Table 4).

There was no statistically significant difference between the groups in terms of postoperative mortality, length of ICU stay, postoperative CVD, AKI, AF, and development of DSSIs.

## DISCUSSION

When all our patient groups were examined, we found no difference in length of hospital stay, postoperative mortality, CVD, AF, and AKI with low LVEF and HbA1c levels. Since diabetic patients were evaluated within themselves, contrary to expectations, we found that the length of hospital stay was longer than those with HbA1c level <7%. In the present study, we observed that AF was the most common complication. The development of DSSI was higher in patients with an HbA1c level ≥7%.

In the literature, more than 40% of patients undergoing CABG are diagnosed with DM^[3]^. When recent studies are examined^[3^,^8]^, they emphasized the superiority of CABG over percutaneous coronary intervention (PCI) for diabetic patients with multiple coronary artery disease. Therefore, CABG is primarly the preferred method of revascularization for patients with DM^[8]^. In open-heart surgery, high blood sugar level in the perioperative period is reported as an independent risk factor for postoperative morbidity and mortality^[2]^. There are some studies^[2^,^3^,^13^,^14]^ reporting that postoperative results are better in patients with strict glycemic control during the perioperative period.

There are different results in numerous studies^[1^,^2^,^9^,^10^,^15^,^16]^ that investigated the relationship between the length of hospital stay and factors such as HbA1c and LVEF. Of these studies, Aydınlı et al.^[2]^ found no statistically significant relationship between high HbA1c levels, postoperative mortality and morbidity, but reported that low LVEF was an independent risk factor for mortality and morbidity. Similarly, Almogati et al.^[1]^ summarized that LVEF would determine the length of hospital stay after CABG, and that there was no relationship between HbA1c and prolonged hospital stay. The results of Knapik et al.^[15]^, Matsuura et al.^[16]^ and Najafi et al.^[9]^ also mentioned similar findings.

Medhi et al.^[10]^ reported that high HbA1c levels were associated with prolonged hospital stay. In addition, Najafi et al.^[9]^ stated that HbA1c may be associated with prolonged hospital stay, but not the prolonged ICU stay. Tennyson et al.^[6]^ suggested that high HbA1c level is a strong marker in predicting mortality and morbidity, regardless of previous diabetic status. In the current study, in contradiction to the previous one, we found that there was no statistically significant relationship between HbA1c level and prolonged hospital stay. This was compatible with the studies by Almogati^[1]^, Aydınlı et al.^[2]^, Knapik et al.^[15]^, Matsuura et al.^[16]^ and Najafi et al.^[9]^.

Although Almogati et al.^[1]^ and Aydınlı et al.^[2]^ found that there was a relationship among low LVEF, length of ICU and hospital stays and postoperative mortality, we could not observe this relationship. We started from the assumption that this situation was due to the difference in the cutoff value (40%) for the LVEF level, determined methodologically in the literature. However, our finding was similar to that of Najafi et al.^[9]^.

Kuhl et al.^[17]^ reported that they could not establish a relationship between HbA1c levels and mortality in patients using insulin. When HbA1c levels are ≥7%, an increase in postoperative complications has been reported^[17^-^19]^. Similarly, Tennyson et al.^[6]^ reported that the risk of mortality quadrupled when the HbA1c value was >8.6%. Beattie et al.^[20]^ reported that HbA1c is a marker of an unchangeable and progressive process, although it shows poor glycemic control^[20]^. Khan et al.^[11]^ accepted the threshold value of 7% in terms of HbA1c. For this value, when they divided the patients into two groups, they found no difference in mortality. On the contrary, they stated that smoking, renal failure and preoperative low LVEF values were associated with increased mortality. Furthermore, in the same study, Khan et al.^[11]^ emphasized that perioperative glycemic control may have a closer relationship with postoperative mortality rather than preoperative HbA1c levels. According to Khan et al.^[11]^, it is widely accepted that patients with uncontrolled diabetes have impaired insulin sensitivity, which requires a higher amount of intraoperative insulin infusion, and stated that patients had a high risk profile due to conditions such as advanced age, the distribution of lesions in the coronary arteries and the need for urgent surgery^[11]^. In the current study, when all patient groups were analyzed, hypertension and CKD were found at a higher rate in diabetic patients and peripheral artery disease in patients without diabetes. We did not find any statistically significant difference between the groups in terms of postoperative mortality, CVD, AF and AKI.

Khan et al.^[11]^ stated that low LVEF could cause prolonged hospital stay, respiratory, renal and vascular complications. In the present study, the rates of CKD and COPD were higher in patients with lower LVEF. However, we found no statistically significant difference between groups in terms of postoperative mortality, CVD, AF, AKI, length of hospital and ICU stays, and development of DSSI.

AF is the most common complication after CABG^[1^,^9^,^10]^. The results of the present study are also in agreement with this finding.

Lazar et al.^[4]^ reported that strict glycemic control is more important in patients undergoing CABG and reduces the development of ischemic cerebral events and mortality. In our study, we found no relationship between HbA1c and postoperative mortality, CVD, AF and development of AKI. Similar results were obtained when diabetic patients were examined within themselves. Our findings were considered compatible with the current studies^[1^,^2^,^11^,^17]^.

Akram et al.^[21]^ used the RIFLE (Risk, Injury, Failure, Loss of kidney function, and End-stage kidney disease) criteria to determine the development of AKI and stated that prolonged CPB and development of AKI are highly interconnected conditions. In support of this, some studies are reporting the link between postoperative AKI and mortality^[21^,^22]^. In the present study, there was no statistically significant difference in terms of development of AKI. This situation was associated with the similarity between XCL and CPB times, and shorter XCL and CPB times in diabetic patients with an HbA1c level <7.

In the literature, there are some reports that investigated the development of DSSI in patients undergoing open-heart surgery^[11^,^23]^. Sevük et al.^[23]^ stated that there was no relationship between high perioperative HbA1c and DSSI, and that there was no increase in the frequency of DSSI development in patients with adequate perioperative glycemic control. Similarly, Kuhl et al.^[17]^ reported that they found no relationship between HbA1c level in perioperative mortality and DSSI development. Contrary to the above, DSSI was seen in patients with an HbA1c level ≥7%. Our findings were consistent with the results by Knapik et al.^[15]^, Halkos et al.^[18]^, Finger et al.^[24]^, and Hadjinikolaou et al.^[25]^.

## CONCLUSION

In conclusion, we found that elevated HbA1c is the most important criterion in infection and hospitalization period, while the relationship between LVEF and diabetes in early in-hospital mortality, prolonged ICU stay and development of postoperative complications has not been determined.

As our study was retrospective, the records and morbidity rates of some patients could not be reached after discharge from our clinic. For this reason, the situations that cause morbidity were not evaluated in our article.

**Table:** 

Authors' roles & responsibilities	
RÖ	Substantial contributions to the conception or design of the work; or the acquisition, analysis or interpretation of data for the work; final approval of the version to be published
AT	Substantial contributions to the conception or design of the work; or the acquisition, analysis or interpretation of data for the work; final approval of the version to be published
HY	Substantial contributions to the conception or design of the work; or the acquisition, analysis or interpretation of data for the work; final approval of the version to be published
GS	Substantial contributions to the conception or design of the work; or the acquisition, analysis or interpretation of data for the work; final approval of the version to be published
HKT	Substantial contributions to the conception or design of the work; or the acquisition, analysis or interpretation of data for the work; final approval of the version to be published
